# Quantifying Patterns of Smooth Muscle Motility in the Gut and Other Organs With New Techniques of Video Spatiotemporal Mapping

**DOI:** 10.3389/fphys.2018.00338

**Published:** 2018-04-09

**Authors:** Roger G. Lentle, Corrin M. Hulls

**Affiliations:** Physiology Department, Institute of Food, Nutrition and Human Health, Massey University, Palmerston North, New Zealand

**Keywords:** spatiotemporal mapping, D-maps, L-maps, area strain rate maps, smooth muscle contraction

## Abstract

The uses and limitations of the various techniques of video spatiotemporal mapping based on change in diameter (D-type ST maps), change in longitudinal strain rate (L-type ST maps), change in area strain rate (A-type ST maps), and change in luminous intensity of reflected light (I-maps) are described, along with their use in quantifying motility of the wall of hollow structures of smooth muscle such as the gut. Hence ST-methods for determining the size, speed of propagation and frequency of contraction in the wall of gut compartments of differing geometric configurations are discussed. We also discuss the shortcomings and problems that are inherent in the various methods and the use of techniques to avoid or minimize them. This discussion includes, the inability of D-type ST maps to indicate the site of a contraction that does not reduce the diameter of a gut segment, the manipulation of axis [the line of interest (LOI)] of L-maps to determine the true axis of propagation of a contraction, problems with anterior curvature of gut segments and the use of adjunct image analysis techniques that enhance particular features of the maps.

## Definitions

**Compliance of the wall:** the ability of the wall of a hollow structure to undergo deformation from increase in luminal pressure. The reciprocal of stiffness.

**D-map:** A spatiotemporal (ST) map based on changes in the diameter i.e., number of pixels between the upper and lower border at an array of locations along the length of a hollow tubular structure over time.

**Phase:** The position of a point in time on a waveform measured in degrees.

**Phase difference or offset:** Difference in in degrees of phase between two waveforms at a particular point in time.

**R-map:** An ST map based on a longitudinal sequence of changes in the distance in pixels from either the upper or lower border of a tubular structure to the edge of a marker or structure that is positioned longitudinally along the center of the profile e.g., a colonic taenia.

**Strain:** degree of deformation induced by an application (or removal) of a force to a tissue such as the gut wall. Positive strain i.e., an increase in distance between two markers, occurs with a stretching (tensile) force and negative strain i.e., a decrease in the distance between two markers, occurs with a compressive force.

**Strain rate:** the rate at which positive or negative deformation takes place.

**Area strain:** the deformation in along the diametric side of square of a tissue in the gut wall multiplied by the degree of deformation of the longitudinal side consequent on the application of a (contractile or other) force.

**Area strain rate:** Rate of change in the area of a square of tissue in the gut wall consequent on the application of a (contractile or other) force.

## Introduction

The use of video spatiotemporal maps to define the timing and site of smooth muscle contraction has rapidly increased over the last decade, such that over 1,500 reports have been published since the late 1990s. Until recently the techniques had been used to quantify movements in various components of the gut. In this context the technique has provided valuable insights into the genesis and control of contractile activity in the gut as well as providing a source of reciprocal illumination in which to view electro-physiological findings. Hence spatiotemporal mapping has proven useful in determining how specific receptors (Abdu et al., 2002) microflora (Collins et al., 2014) genetic mutations (Roberts et al., 2008) or pharmacological interventions (Bogeski et al., 2005; Spencer et al., 2013; Schreiber et al., 2014) alter motor activity in the relevant organ. Moreover, the concurrent use of longitudinal and radial mapping allows for resolution of the contributions of the circular and longitudinal muscle (Lentle et al., 2008) and the spatial resolution of the outputs of the neural circuitry (Lynn et al., 2005). Further, the use of recently developed area ST mapping techniques can allow the pattern of growth and propagation of fronts or areas of smooth muscle contraction across the surface of large hollow organs such as the bladder to be mapped (Lentle et al., 2015) and the effects of tone to be surveyed (Lentle et al., 2016).

**Figure 1:**
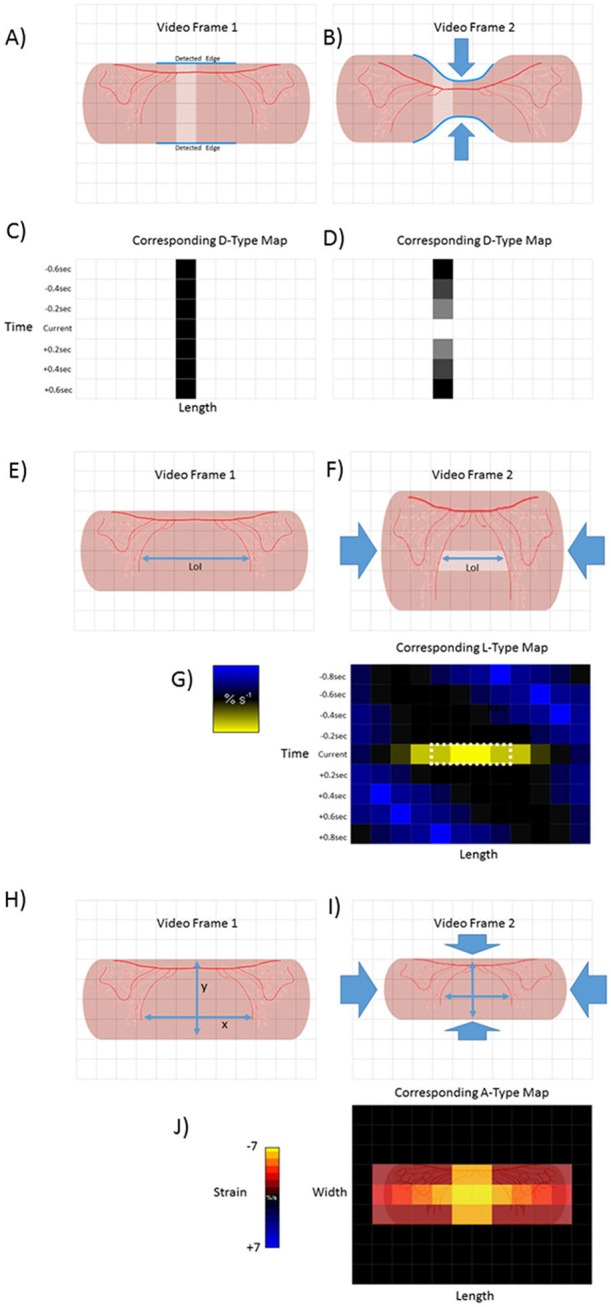
Basic methodologies of D-type, L-type and A-type video-spatiotemporal mapping techniques. D-type ST maps **(A–D)**. The upper and lower edges of a structure (in this case a pink tubular segment of gut) are detected (blue line) and the number of pixels counted between the edges of successive columns (pale area). The totals are then color coded for each column within each frame. Hence the dark square in the in time series of D map below frame 1 **(C)**. Circumferential contraction (frame 2) causes reduction in diametric profile and corresponding pixel total (light square in time series of D-type map below) **(D)**. L-Type ST maps **(E–G)**. The line of interest (LOI) that is to be used as a datum point for quantification of length-wise movement in successive frames is chosen (blue line). The color coded magnitudes of changes in distance (strain) between distinctive features (in this case red vascular markings) on successive video frames (strain rate) are plotted for each pixel along the length of the LOI (in this case shown only for one set of distinctive features between video frame 1 and 2) A-type ST maps **(H–J)**. The diametric and lengthwise changes in distances between distinctive features (in this case red vascular markings) in successive frames are determined over each component pixel over a defined area within the edges of the structure. The derived diametric and lengthwise changes within each pixel in successive frames for are then multiplied to give area strain rate. The color coded values for strain rate then overlaid onto the corresponding pixels of the video frame to produce a map of area strain. Hence map **(J)**, shows the hypothetical two dimensional result of a contraction at a single point.

A particular advantage of the spatiotemporal mapping method is that it avoids the insertion or application of monitoring devices into, or onto, the surface under study. The lack of any requirement for surface contact during ST mapping and consequent low risk of contamination could make the technique potentially useful during laparoscopy provided that small video cameras with sufficient image definition are available. Hence, ST mapping of laparoscopic video sequences could be useful in mapping the contractile outcome of conditions such as diabetic gastroparesis that are known to be accompanied by adverse electrophysiological states such as slow wave dysrhythmias and re-entrant rhythms (Chen and McCallum, 1992). Spatiotemporal mapping can provide a stream of real time data regarding the frequencies, speeds and directions of propagation, amplitudes and durations during progression of a contraction through the walls of a hollow tubular or sacculate structures (Figure 1) that can subsequently be incorporated into computational fluid dynamic models to assess their effect on the mixing and onflow of their contents (de Loubens et al., 2014).

Whilst alternative methods such as water perfused (Di Lorenzo et al., 2002) and fiberoptic (Dinning et al., 2014) catheters and barostats (Ohe et al., 1994) can be used to directly assess changes in the pressure within the lumen of such structures and thus to infer motility, they generally do not provide sufficient spatial or dimensional resolution to enable the mixing regime to be accurately defined. Thus, the use of high resolution fibreoptic catheters that record local changes in lumen pressure at intervals of around 1 cm along the lumen (Dinning et al., 2014), have similar limitations to those of early spatiotemporal mapping methods based on widely spaced markers (Hennig et al., 1999), notably being prone to aliasing. Hence a number of types of contraction have short territories and frequencies that are not amenable to resolution with such spacing e.g., haustral boundary contractions in the colon which occupy <0.25 cm of the wall (Chen et al., 2013). Again, the radial positioning of fiberoptic catheters within the gut lumen cannot be precisely defined or controlled. Currently the differences in the results from fiberoptic high resolution manometry (Dinning et al., 2014) and from water perfused catheter techniques (Rao et al., 2001; Camilleri et al., 2008) from those obtained by D type spatiotemporal mapping (Lentle et al., 2008; Dinning et al., 2012; Costa et al., 2013a; Chen et al., 2016) raise questions regarding the nomenclature of contraction. Hence for example there is no clear evidence from manometry of the existence of high frequency ripple contractions in the human colon that are similar to those observed in rats and rabbits (Costa et al., 2013a; Chen et al., 2016) despite the presence of a similar pacemaker system in humans (Rae et al., 1998) to those in animals that generates oscillation in myenteric potentials of a higher frequency than those of slow waves.

## The development of video spatiotemporal mapping techniques

In this section we describe the development of the various types of video spatiotemporal map and the advantages and disadvantages of each method. The progenitors of video spatiotemporal mapping techniques, described 18 years ago (Hennig et al., 1999) were based on changes in the outline of the organ (Figures 1A,B), and in the positions of regularly spaced markers, generally in the longitudinal dimension, which latter can be regarded as the mapping of strain (Hennig et al., 1999).

Ultimately, video spatiotemporal mapping comprises the comparison through time of the spatial position of an object or of distinctive features of the object. Later workers, notably Cannon (1898), compared the profiles of a succession of images that were drawn, filmed (Alvarez and Zimmermann, 1927; Tasaka and Farrar, 1969) or X-rayed (Bowditch, 1897; Cannon, 1898) to characterize the contractile behavior of various segments of the gut.

The advent of video recording permitted the motion of distinctive features in an area under investigation to be followed through a stream of successive images taken at a uniform intervals whilst the development of image analysis techniques (Russ, 2006), allowed search algorithms to be successively applied to each frame to identify edges (Sobel, 1970), with subsequent plotting of the distances between them at successive points along the length of the segment of gut, a technique termed diameter or D type mapping. This technique was originally applied to the small intestine of the rat maintained *ex vivo* (Benard et al., 1997) and subsequently to the stomach (Berthoud et al., 2002; Lentle et al., 2010), small (Hennig et al., 1999; Lentle et al., 2007), and large (Gwynne et al., 2004; Dinning et al., 2008; Lentle et al., 2008; Hennig et al., 2010a) intestine from a range of species maintained *ex vivo* as well as *in vivo* (Janssen et al., 2014).

Whilst these video spatiotemporal mapping techniques allowed changes in the diameter and length of the walls of hollow tubular structures such as the gut to be quantified, the subsequent development of strain rate mapping i.e., L-type ST maps (Lentle et al., 2007) and area strain rate (Lentle et al., 2015) i.e., A-type ST maps allowed local changes in length and in area to be determined and plotted over time with higher resolution. This allowed the site of contraction of circular and longitudinal components of the *tunica muscularis* to be directly identified at all points within the borders of the image. Further, the rhythmic sequence of longitudinal and circumferential components could be simultaneously mapped along a user-defined line of interest (LOI) located at a particular site on the intestinal wall (Janssen et al., 2014).

### D-Type video spatiotemporal maps

#### Method and interpretation

D type spatiotemporal maps are based on changes in the diameter of a segment or organ over time (Figures 1A–D). Typically, edge detection algorithms (Russ, 2006) are used to search for and delineate the upper and lower edges of a tubular length of gut that is positioned with its long axis parallel to the upper and lower borders of the video frame (Figure 1). Edge detection algorithms, such as those described by Sobel (1970), Kirsch (1971), and Frei and Chen (1977) work well with ST mapping of preparations that are maintained *ex vivo* in a situation when there is good contrast between the optical characteristics of the preparation and the background (indeed many operators ensure that there is a uniform black or white coloration on the background surface of the organ bath).

**Figure 2:**
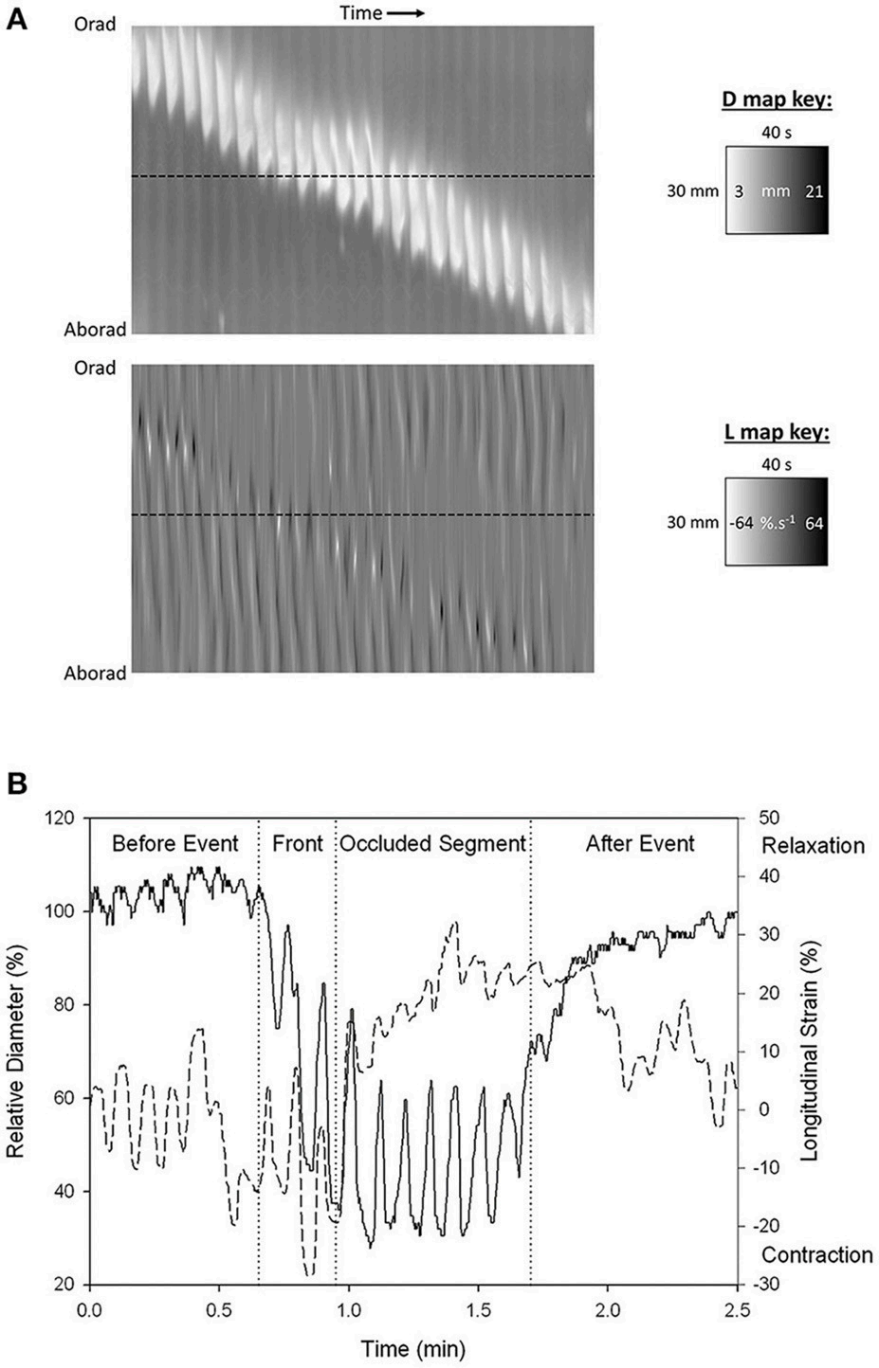
Synchronous **(A)** D-type (diameter type) ST map (left) and L-type (right) (linear strain rate) ST maps and graph of transects **(B)** showing progression of a peristaltic contraction though a segment of possum ileum. **(A)** Lighter areas on the D-map indicate sites along the length of the organ where the diameter is reduced and darker areas sites where the diameter is increased. The thickness of the band measured horizontally is the duration of the contraction. The thickness of the band measured vertically is the length of the organ that is undergoing contraction at any given time. The angulation of the band to the horizontal (time) axis is proportional to the velocity of propagation of the constricted area. Note the component vertical “stripes” on the D map which indicate rapid activation of successive regions of smooth muscle occurring at the slow wave frequency. Note also the darker shading i.e., distension in advance of the propagating lighter band than that in the rear showing that lumen contents are being propelled in advance of the constriction. Lighter areas on the L map indicate sites of negative longitudinal strain rate i.e., constriction in the longitudinal dimension. Events at the horizontal dotted line, placed at the same site in the two maps (**B** below), indicate that longitudinal shortening occurs in advance of the circular constriction **(B)** Coordination between D (solid line) and L (dashed) maps during a peristaltic event. Values were taken from the horizontal line delineating a particular site on the synchronous D and L maps. Contractions are seen to be 180° out of phase prior to the peristaltic contraction and in phase during the contraction. The amplitude of circular contraction on the D map increased during the peristaltic event. Figures adapted from Lentle et al. (2007).

The total distances in pixels between the two edges are determined for every pixel column along the visible length of the preparation (Figure 1). The totals for successive columns are each then plotted as a shade or color coded total per column so as to form a pixel row across the D map (Janssen and Lentle, 2013). The procedure is repeated for each frame and successive rows each apposed below the preceding row so that the vertical distance of any row from the first in the series is proportional to the cumulative number of frames and hence to the elapsed time (Figures 1, 2). Thus any site along the length of the gut where the diameter is relatively reduced will have a lower low pixel sum and be of a lighter (or darker) shade on the D-map than one at a site that is distended which will have a higher pixel sum. On such a map a propagating peristaltic contraction will generate a band of lighter (or darker) pixels of the D map that shows lengthwise progression in successive rows (Figure 2). The duration of the contraction at any given point will be shown by its breadth on the time axis and its lengthwise extent by its breadth on the distance axis. Similarly, the speed of its lengthwise propagation will be directly proportional to the angle of the (lighter or darker) band to the time axis, greater angles indicating faster progression. Further, when a regular succession of contractile events occurs, their frequency can be determined at any point on the length of the gut component from the number of lighter bands that transect a line of known duration drawn from that point on the ST map parallel to the time axis. Again D-type ST maps are useful in identifying points along the length of the intestine at which the frequencies of contractions change (phase dislocations), which are seen on the D map as points of fusion or bifurcation of individual propagating contractions. The sites and timings of these changes have been related to the coupled oscillator theory of propagation (Parsons and Huizinga, 2015) which hypothesizes that the spontaneous oscillations in neighboring interstitial cells of Cajal (ICC's) become entrained within the limits of their respective inherent frequencies. Hence when the difference in the inherent oscillation frequencies of neighboring ICCs reaches its limit there is a sudden change in the frequencies of their slow waves and corresponding contraction frequencies.

Currently there is no consensus regarding which axis of ST maps is displayed vertically and which is displayed horizontally. However, the convention used by a number of workers in which the length axis is vertical axis and time horizontal a (Dinning et al., 2012; Costa et al., 2013a; Chen et al., 2016) may make the visualization of rate easier as the slope described by the contractile event is more readily seen to be proportional to its rate of propagation and is similar in orientation to that used in most reports of catheter studies.

**Figure 3:**
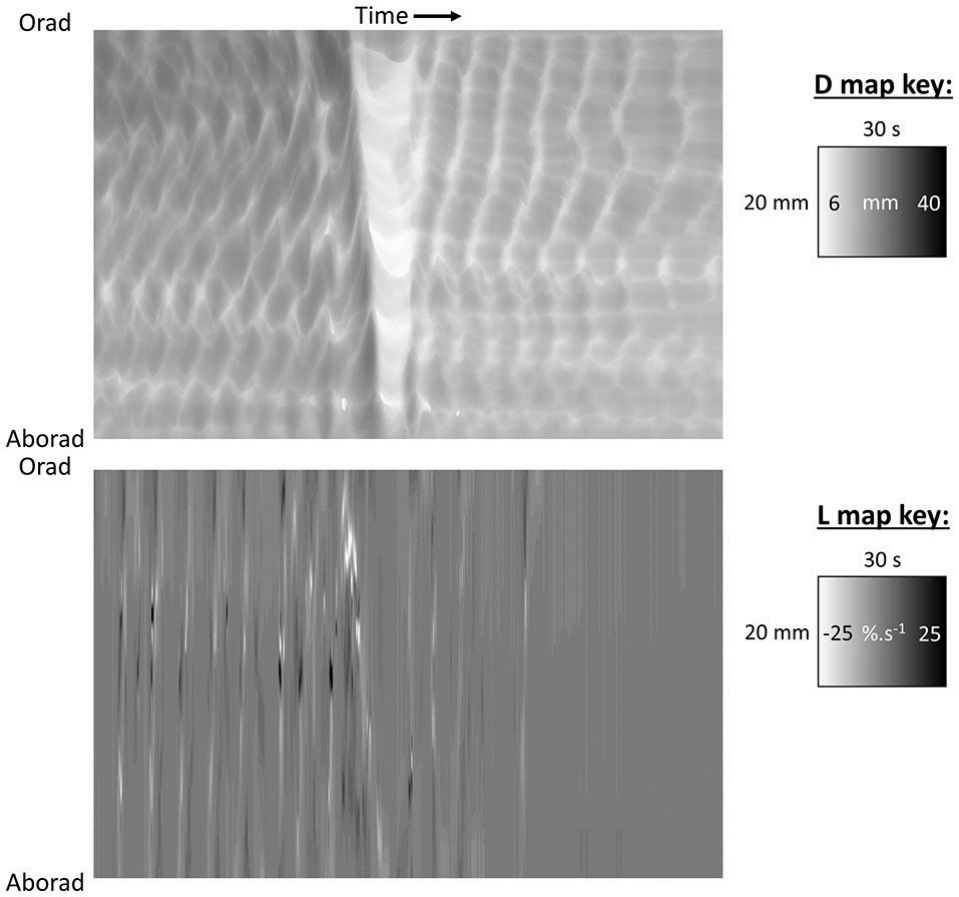
D-type ST map **(Top)** and L-type ST maps **(Bottom)** of a mass peristaltic event traversing the proximal colon perfused with saline. The triangular white area on the D-map indicates a diametric constriction from mass peristalsis rapidly propagating from proximal to distal along the organ with a darker area from distension of the lumen by distally displaced contents traveling in advance. Note that the constriction propagates from proximal to distal but relaxation progresses from distal to proximal as can be seen by the differences in the slopes of the onset and offset of the event and the consequent triangular configuration. The angled track of narrow white areas (-ve strain rate) on the L map **(Bottom)** indicate a sequence of short-lived longitudinal constrictions occurring in advance of the diametric constriction of peristalsis. Note that more regular and more rapidly and extensively propagating longitudinal constriction (likely fast phasic contractions) and column of episodic localized radial constriction (likely ripple contractions) occur at times when there are no peristalses. Figure adapted from Lentle et al. (2008).

Darker areas on D-type ST maps, signifying relative distension of the lumen, can provide information regarding the movement of contents and their influence on the pattern of radial constriction. Hence, in the colon, a darkened zone propagating in advance of a diametric constriction may indicate displacement or accumulation of contents (Figure 3; Lentle et al., 2008). Further, the triangular configuration of the (white) area of diametric constriction on the D-map indicates that the constriction persists at all sites until it has propagated along the entire length of the preparation whereupon relaxation, signified by the darker area, progresses from the distal to the proximal end. Such a contractile configuration would likely maximize the emptying of the viscid contents of the colon by preventing backflow.

#### Drawbacks

D type spatiotemporal maps have several shortcomings. Firstly, in summing the total number of pixels at a given point along the length of the gut, D maps sum the cumulative effects of smooth muscle contractions that occur at all of the sites across the diametric transect and do not describe the relative contribution of any localized contractions at particular points across the diameter. Hence for example while it is reported that there is anisotropy in the degree of contraction between the mesenteric and anti-mesenteric sides of the small intestine (Lentle et al., 2012), D maps cannot identify the sites of such differences. This shortcoming is a particular problem at sites where the muscles associated with the upper and lower edges behave autonomously so that the profiles of the upper and lower borders of the gut do not correspond, for example during ripple contractions in the haustrated colon (Lentle et al., 2008). Hence for example, at any given point on its length, the pixel sum on a D map that reflects the overall reduction in diameter will be influenced by the extent to which the changes in edge profiles, which reflect ripple contractions in the upper and lower halves, are synchronized. This problem cannot be circumvented by incorporating an algorithm that determines the midpoint between the upper and lower borders of the gut and determines changes in each separate half (radial segment) as, if the two edges are moving independently or differently, both will affect the position of the midpoint at that instant. However, this problem may be countered in cases where there is a distinctive longitudinally disposed structure on the cylindrical surface of the gut wall. With appropriate rotation, the edges of such a structure can be positioned along the midline between the upper and lower edges of the image and used to subdivide the diametric dimension of the structure. Hence for example when the *taenia libra* is positioned midway between the upper and lower borders of the colon this structure provides a consistent basis on which to subdivide the image into upper and lower radial halves allowing a separate “R” map to be plotted for each (Lentle et al., 2008). The use of this technique can allow the movements of colonic haustrae in each radial half of the preparation to be separately examined and subsequently compared (Lentle et al., 2008).

Even in situations where contractions within the diameter of a tubular element of the gut, such as the small intestine, arise synchronously in the upper and lower halves, problems can arise with assessing such characteristics as its speed of propagation. As stated hitherto, the velocity with which a contractile front propagates along a tubular length of gut is generally considered to be proportional to the angle of the track of the contraction with the axis defining time. However, if the contraction involves only a limited region of the visible intestinal wall, it is possible that it can propagate over a number of possible curved trajectories that are not aligned with the lengthwise axis of the organ, for example in a spiral course (Wang et al., 2004; Laforet et al., 2013). Hence the true velocity of propagation will be greater than that shown on the D-map as the distance covered will be greater than the overall length of the segment.

**Figure 4:**
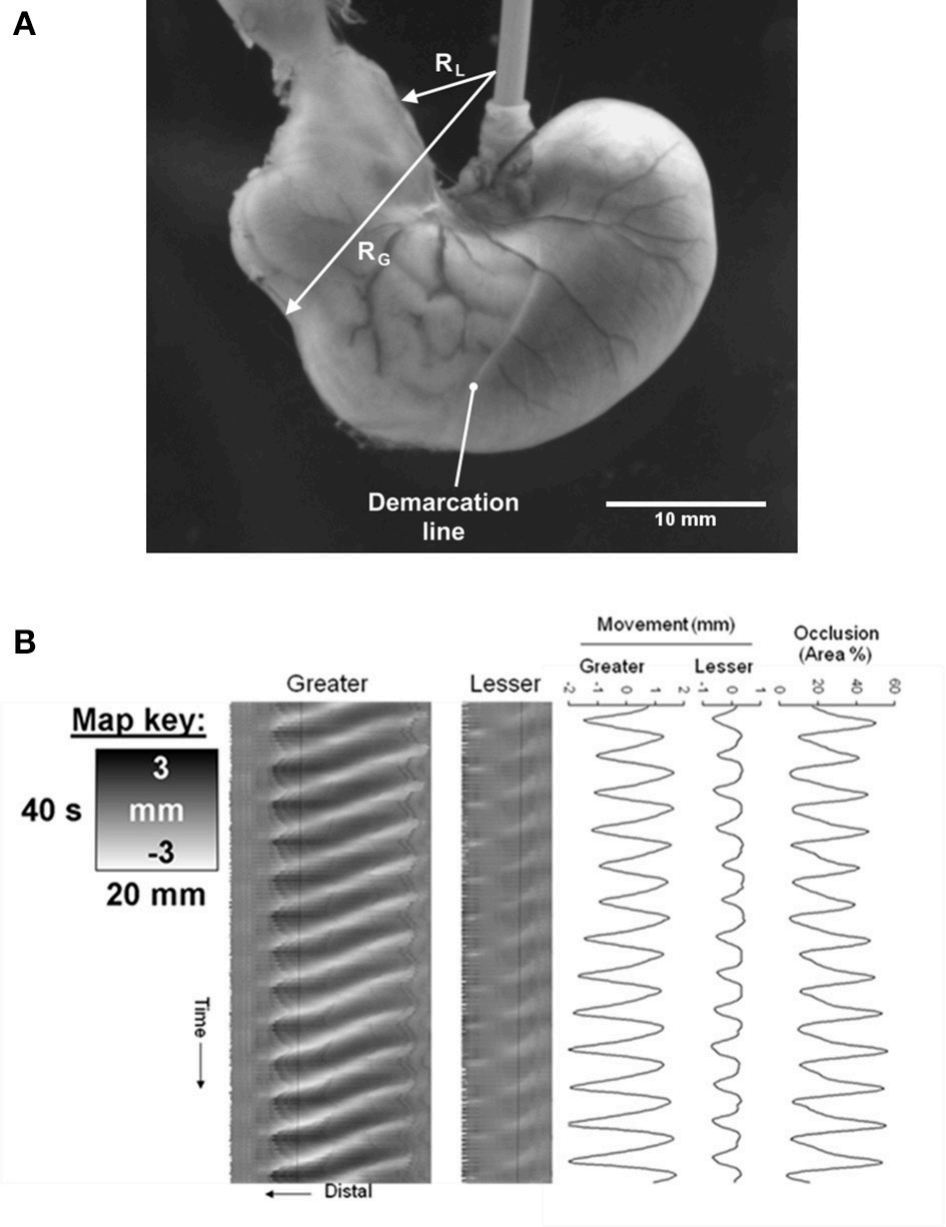
D-type ST map of transit of gastric peristalses along an arc based near the greater curvature (abscissa) and centered at the anterior limit of the esophageal junction. **(A)** Diagram showing the basis of the arc of curvature from which spatio temporal maps are derived with the cumulative angle of deviation on the horizontal axis with the intensity proportional to the distance along the line between the lesser (RL) and greater (RG) curvatures with lighter shading for longer and darker shading for shorter distances. The scaling of the raw maps can be converted to linear distance from the pylorus to enable linear velocity to be quantified. **(B)** D-type ST map showing the rhythmic sequence of angled lighter bands of radial constriction from regular antral peristaltic events. The uniform thickness of the bands indicates they are of constant duration regardless of their position with respect to the pylorus. The uniformly linear trajectory angled toward the pylorus (on the left) indicates that their velocity of propagation is uniform along the arc of the LOI. Note that the darker area lying to the right of each propagating contraction is from dilatation from retropulsion of contents and that the extent of this distension decreases near the pylorus presumably from increased thickness and decreased compliance of the distal walls. Figure adapted from Lentle et al. (2008).

A similar problem arises when assessing the direction in which contractions propagate through gut components that are not tubular in shape. Hence when the passage of antral contraction waves through the stomach of the guinea pig is mapped across its three constituent sections (the proximal, middle and distal components of the stomach), with the pixel columns orientated at right angles to the longitudinal axes of symmetry in each section, the trajectory of the contraction on the D-map is of a sigmoidal configuration with respect to time and varies in duration (thickness) (Berthoud et al., 2002). On the other hand, in another analyses of the transit of antral contraction waves in the rat stomach, when the pixel columns for the D maps were positioned orthogonal to an arcuate axis of symmetry centered at the anterior limit of the junction of the esophagus with the cardia (Figure 4A), the trajectory of propagation of antral contractions on the ST map was a straight line of near uniform breadth (Lentle et al., 2010) indicating that the velocity of propagation was constant as was the duration (Figure 4B). The principal of parsimony (Occams razor) would favor the latter situation where the velocity of propagation is invariant and duration constant. Hence plots of D-type ST maps based on principal axes of various geometries can thus be used iteratively to determine the direction of propagation of contractions across the surface of a non-tubular organ, assuming that the map will be linear when the geometry is aligned with the principal axis of propagation. Note that this method is broadly akin to the use of different lines of interest (LOI) on which to base L maps (see below).

A third problem with D type ST mapping is “spurious” movements i.e., movements not due to active muscle contraction in the dimension under examination but from the effects of a contraction on the adjacent gut wall. Hence a localized contraction of circular or longitudinal muscle occurring at a given site on a length of gut may cause the adjacent wall to be drawn toward it or to be pushed away. This may be due in part to the incompressibility of the tissue that is undergoing contraction. Hence, the contraction of the long axes of circularly orientated myocytes in the circular muscle layer of the gut wall will cause a simultaneous increase in their width as their fluid contents are incompressible, generating a lengthwise expansion that will displace adjacent uncontracted circumferentially orientated myocytes longitudinally (Gregory and Bentley, 1968). Such movement may be mistaken for relaxation of the longitudinal musculature. Similarly, myocytes that are orientated longitudinally will expand circumferentially whilst contracting, generating an action that may be mistaken for circumferential contraction. It is noteworthy that these movements may each in turn be transmitted to the other muscle layer. Hence for example circumferential lengthening, generated by circumferential contraction, may generate “passive” lengthening of the associated longitudinal muscle. Such “passive mechanical interactions” have been implicated in studies of interactions between contractions in the longitudinal and circular muscle layers of the distal colon of the guinea-pig (Spencer et al., 1999; Spencer and Smith, 2001). An alternative cause of “spurious” movement, is that which results from the inherent elasticity of the gut wall which may draw any tissue that is undergoing active relaxation or accommodation toward it. At either event, a phasic contraction occurring in a length of gut will generate a change the longitudinal disposition of the constituent and adjacent pixel columns in relation to the proximal and distal ends of the gut causing their column “address” within the video frame to change, a process that we term “shunting.” These problems can occur both *in situ* and *ex vivo* but can become particularly severe when lengthy sections of gut are mapped, for example an entire colon. In *ex vivo* preparations these problems may be exacerbated by sagging i.e., a tendency for the central region of a segment to sink within the organ bath during relaxation and rise during contraction. Whilst it is not possible to entirely eliminate such sagging, its magnitude may be reduced either by positioning a lengthwise a plastic rod within the intestinal lumen or by supporting the intestine on a shallow cradle termed a kit-kat (Parsons and Huizinga, 2015). Alternatively, where practicable, such sagging may be reduced by limiting the length of the *ex vivo* segment that is mounted in the organ bath.

The generation of D type ST maps from segments of gut that are maintained *in situ* within the abdominal cavity is often complicated by impairment of the detection of the edges of the section under study. Hence for example, when studying the intestine at laparotomy, the partial overlaying of one loop of gut upon another reduces the surrounding area of contrast compared to that in an organ bath. Whilst viable loops can be isolated and maintained on (white or green) saline moistened swabs at laparotomy (Janssen et al., 2009), this procedure may generate additional tension on each end of an isolated segment that may exacerbate shunting.

A particular advantage of D-type over L-type ST maps is that they avoid problems that arise from anterior curvature of the surface under study. This curvature results in a gradation of the angle at which the surface is viewed within the video frame which influences the magnitude of any surface displacement caused by a given contraction. When gut segments such as the small intestine are mapped on a basis of surface displacement i.e., undergo L-or A-type mapping (see sections below), the curvature is generally insufficient to produce significant reduction in the apparent magnitude of a contraction except at the extreme periphery. However, when larger structures such as the urinary bladder are mapped using L- or A-techniques, the sites where there is significant reduction in magnitude will extend for greater distances from the edges. Hence D-type maps are better able to accurately determine the magnitudes of movements around the edges of such structures than are L-type or A-type ST maps.

### Video spatiotemporal maps of strain and strain rate

#### Method and interpretation

A number of workers have used a method for assessing local changes in length based on the distances between sets of evenly spaced markers i.e., strain (Hennig et al., 1999; Schreiber et al., 2014). However, as with D-type ST maps, the distance between any two markers will be that from shortening of smooth muscle at various sites, minus that from lengthening at other sites from active relaxation and from passive elastic stretching. However, the plotting of the rate of change in strain i.e., strain rate, between multiple randomly and finely distributed marker points, along a user defined LOI (Figures 1E–G) can distinguish local areas of negative strain rate that result either from contraction of smooth muscle or from recovery after stretching, from areas of positive strain rate that result from muscular relaxation or from passive stretching, and from sites where neither contraction or elastic changes are occurring where strain rate is zero.

Anatomical markers that are distributed along the LOI, such as distinctive junction points in vascular arcades, can be used as reference points along the LOI. Alternately, at sites where such natural markers do not occur, a variety of particulate inert substances such as India ink (Melville et al., 1975), silk knots (Hennig et al., 1999), dots of soot (Lammers et al., 2001), and 100 μm flecks of glitter (Hennig et al., 2010b) have been applied to the gut wall as artificial markers.

Early researchers used changes in the distances between successive artificial markers that had been placed at regular intervals along the LOI to estimate longitudinal shortening or lengthening (Melville et al., 1975; Hennig et al., 1999). Recently, more sophisticated algorithms based on cross-correlation (Janssen and Lentle, 2013) have been developed that do not depend on determination of changes in distance between equally spaced markers. Briefly, the movement of a reference point within a characteristic pattern of randomly distributed markers is detected in a 21 × 21 pixel square surrounding it. This dimension is adequate for use with vascular markers when these are evident e.g., in the small intestine *in vivo* (Lentle et al., 2007) or when randomly placed particles of carbon black are applied to the surfaces of less vascular structures (Lentle et al., 2015). The function describing the relationship between the position of a reference point on the image pattern within that square, and that within a displaced square in the subsequent frame, is given by:

(1) $$\text{C}(\text{x},\text{y})=\sum_{\text{ i }=\;-10}^{+10}\sum_{\text{j }=\;-10}^{+10}{\left(\left(\text{P}(\text{i},\text{j})-\mu_{\text{P}}\right)-\left(\text{Q}(\text{i}+\text{x},\text{j}+\text{y})-\mu_{\text{Q}}\right)\right)}^{2}$$

where C(x,y) corresponds to a displacement of x & y pixels between the current and subsequent frames. P and Q are individual pixel intensities from the current and subsequent frames respectively, while μ_P_ and μ_Q_ are mean pixel intensities for the 21 × 21 pixel squares in these frames. A simpler function based on the sum of the absolute difference in pixel intensities has also been used (Huizinga et al., 2010) but our experience is that this is not a critical factor.

The function is evaluated over a range of integer x and y values that are selected to cover the maximum displacements observed between frames, typically with gastrointestinal smooth muscle around -15 to +15 pixels. The minimum of this function represents the movement in pixels of a point at the center of the square between the successive frames, and hence the local velocity, as the frames are captured at regular time intervals, typically 15 per second (Jähne, 2004). Earlier work simply searched for the pixel displacement with the lowest function value (Lentle et al., 2007; Huizinga et al., 2010). However, the author and co-workers have refined the technique to determine the minimum value by fitting a 2D cubic spline to the cross-correlation data and calculating the minimum value on the fitted spline so as to give the displacement in real numbers, i.e., fractions of a pixel.

Importantly, these methods defeat shunting as the identity of a particular region of the wall is marked by a distinctive local pattern of randomly distributed marker points. However, the cross-correlation mapping technique can have difficulty evaluating slow tonal changes such as the increase in caecal length associated with receptive relaxation (Lentle et al., 2007). This is due to the algorithm determining long term change by cumulatively summing the many small changes that occur between sequential images over that time, a process that tends to accumulate errors over long periods of time. This can be avoided by less frequent sampling of the video frames so that the time interval between successive frames is increased.

The fact that strain rate mapping is able to distinguish contractile states within surfaces gives it several practical advantages over D mapping. Firstly, the fact that it does not depend on the identification of edges, allows it to be used to map localized sites that are distant from the edges of the organ, obviating the need to visualize the entire organ. This attribute could be particularly useful in analysis of laparoscopic video data so as to detect propulsive dysrhythmias in sites where loops of bowel adjacent to the area under study would confound edge detection. Secondly, given that the site of the LOI can be chosen by the operator, a number of L maps based on a sequence of longitudinal LOIs that are positioned longitudinally at various points across the radial dimension of the segment, can be prepared from the same video and the patterns of longitudinal contraction along the various LOIs quantified and compared. The use of this technique has allowed the pattern, amplitude and timing of longitudinal (pendular) contractions at mesenteric and ab-mesenteric sites on the duodenum to be compared and sites of differences identified (Lentle et al., 2012). Again, as with D-type ST maps, the preparation and comparison of a range of L maps based on linear and non-linear LOIs orientated at various angles is useful in determining the principal direction of propagation of a patch contraction on the surface of a sacculate structure such as the bladder (Lentle et al., 2015). As discussed hitherto, the LOI yielding the greatest amplitude and most consistent slope will generally be aligned with the true direction of propagation (Lentle et al., 2015).

**Figure 5:**
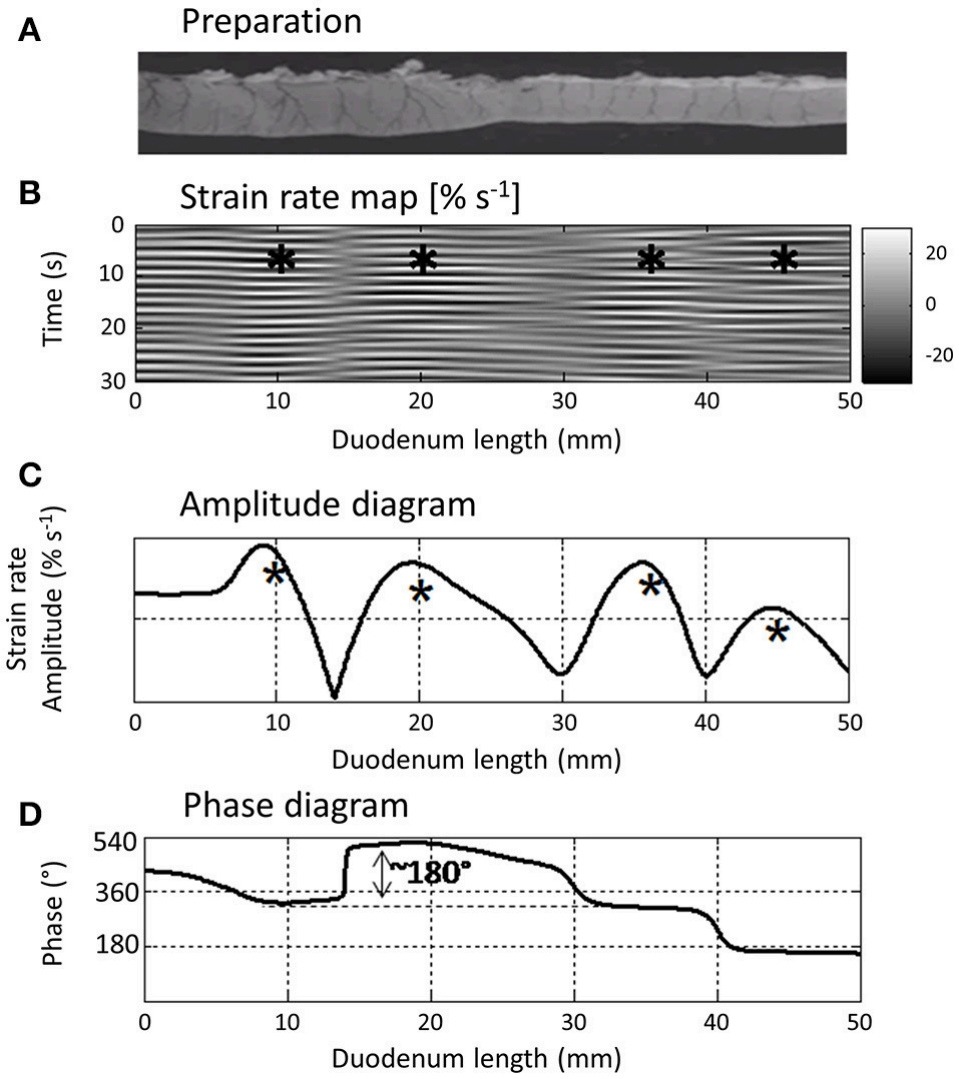
L type ST Map **(B)** showing temporal variation in longitudinal components of segmentative contractions in the proximal duodenum **(A)** of the rat with concomitant variation in amplitude **(C)** and phasic relationship **(D)** over successive contractions. The regular sequences of (white) short-lived (limited vertical thickness) rapidly propagating (near horizontal) longitudinal contractions and following (dark) short-lived dilatation recur at similar sites along the duodenum and form a series of columns of varying length on the L map. The asterisks show the regions in which contractions tend to recurrently peak within each column which the authors term “domains” and the phase diagram the temporal relationship between the cyclic longitudinal contractions within each domain. Hence for example, the peak in amplitude (expressed as a percentage increase in the minimum strain in that domain) of the first marked column occurs during relaxation in the second marked column i.e., is 180° out of phase with the second. Figure adapted from Lentle et al. (2012).

**Figure 6:**
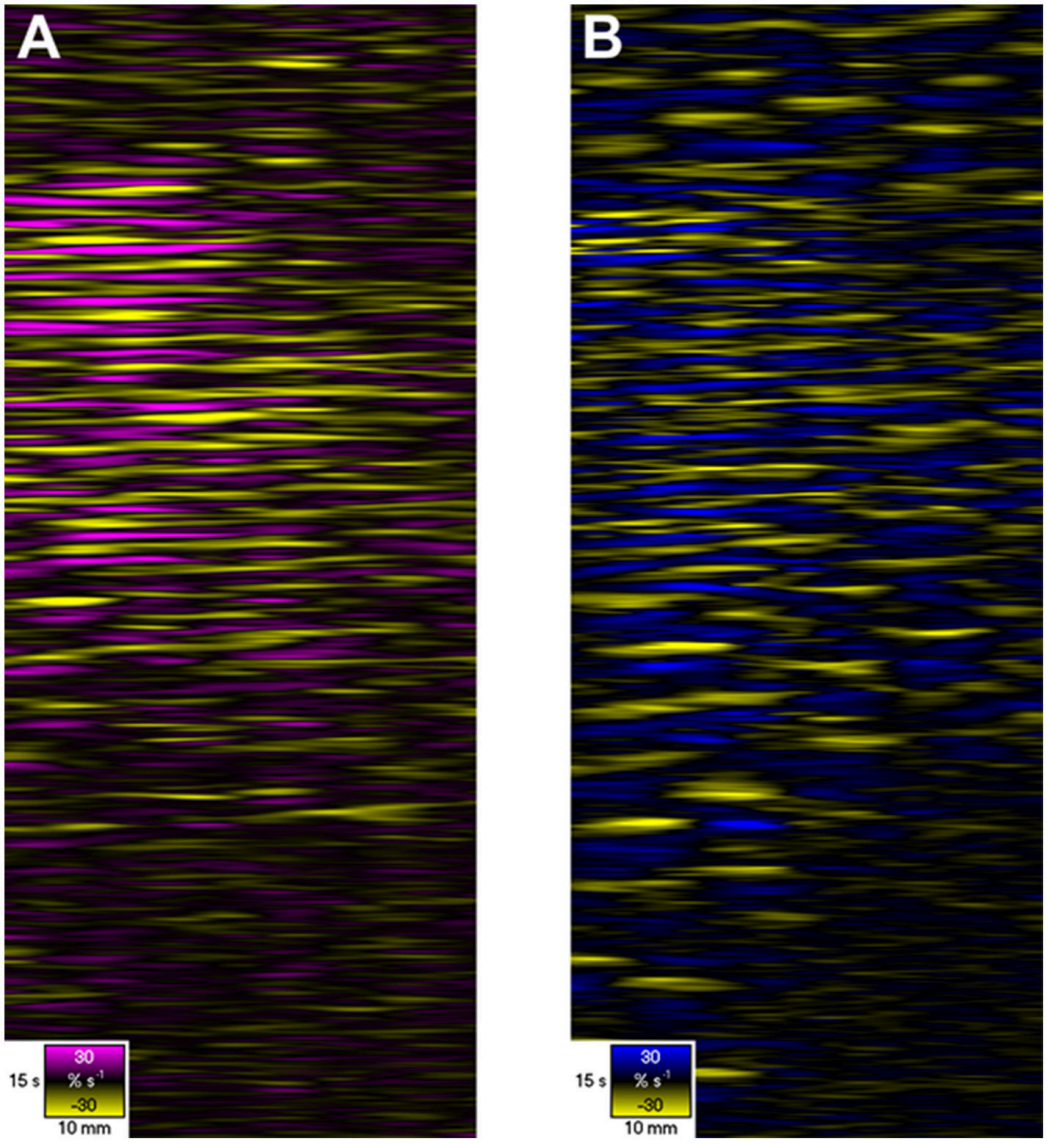
D- and L-type ST Maps showing variation in circumferential **(A)** and longitudinal **(B)** strain rate in the terminal ileum of the pig *in situ* at laparotomy, before and after dosage with remifentanyl. The markers are the vascular arcades which are prominent *in vivo*. Areas of circular **(A)** and longitudinal **(B)** contraction (–ve strain rate) are shown in yellow and areas of circular and longitudinal relaxation in purple and blue respectively (note color codes and magnitudes at the base of each map). Note that the contractions i.e., the discrete patterns of negative circular **(A)** and longitudinal **(B)** strain rate typical of segmentation (Figure 5) in the upper region of the D- and L-type STmaps briefly become aligned i.e., in staggered phase, during the period after the administration of Remifentanyl. Hence the contraction appears to propagate along the length of the ileum. Figure adapted from Janssen et al. (2014).

The high degree of resolution obtained when mapping strain rate along an LOI allows minor changes in the pattern of contraction through time to be distinguished. Hence, the use of L type ST maps has allowed the temporal changes in the disposition of small areas of stationary longitudinal contraction that occur during pendular movements and their phasic relationship to each other to be distinguished, both in the duodenum (Lentle et al., 2012; Figure 5) and in the ileum (Janssen et al., 2014). Further, the technique is capable of distinguishing subtle changes in the pattern of contraction *in vivo*, such as the commencement of coherent distad propagation in a static array of segmentative or pendular contractions when pro-kinetic agents such as remifentanyl are given (Janssen et al., 2014; Figure 6). Similarly, those effects of pharmacological agents which aid in the distinction of myogenic from neurogenic contractions (Lentle et al., 2012).

The ability of strain rate mapping to accurately determine the site, speed of propagation, duration and frequency of cycles in strain rate in a succession of events over a set period of time enables sequences of real time data to be incorporated into mathematical models that assess their effects on wall movement and mixing and transport of the lumen contents (de Loubens et al., 2013, 2014). Moreover, the veracity of the model may subsequently be checked by comparing the predicted mixing outcome with that determined from dilution of a bolus of tracer dye perfused through the preparation over the same time period during which the map was derived (de Loubens et al., 2014).

#### Drawbacks

L-type ST maps may underestimate the amplitude of strain when LOIs are orientated on curved surfaces, for example when the LOI is orientated at right angles to the long axis of a tubular structure such as the intestine. In areas at the periphery of a large spherical structure where angulation of the surface is high, strain rate may be significantly underestimated. In such situations the problem of distortion at the edges of the image can be avoided by the use of multiple cameras, located in such positions that the central parts of the various images cover all aspects of the organ. Alternatively it may be possible to determine the curvature of the surface on which the LOI is located directly with a laser profiler (Hennessy et al., 2002; Strobl et al., 2004) and mathematically weight the strain rates along the LOI accordingly.

### Composite plots and plots derived from video spatiotemporal maps

The positioning of a vertical line at a given lengthwise point on D or L type ST maps, and the recruitment of pixel data from the column coincident with that line, can provide quantitative information regarding component frequencies. Fast Fourier transformation of ST data is commonly used to identify and quantify these frequencies (Neal et al., 2009) and enable their quantitative comparison before and after the application of various pharmaceutical agents (Roberts et al., 2010).

Where a contractile pattern such as peristalsis contains both longitudinal and circumferential components, the judicious use of synchronous vertical ST transects to show the phasic relationship between the two components at a particular point along the length of the preparation (Figure 2B) can shed addition light on their causation. Hence in synchronous D-type and L type ST maps of peristalsis, a forerunning band of negative longitudinal strain rate and a following region of circumferential contraction can be distinguished (Lentle et al., 2007). Comparison of their synchronous vertical (temporal) transects taken at the same site (Figure 2B) showed that the onset of the longitudinal shortening component was synchronous with that of circular contraction presumably reflecting synchronous neural signaling.

Again, where L- type ST maps of cylindrical structures such as the small intestine show longitudinally distributed arrays of stationary contractions within successive domains, similar comparisons of a sequence of vertical map transects can distinguish their phasic relationships and relative amplitudes (Figure 5; de Loubens et al., 2013, 2014).

In respect of D maps, it is noteworthy that, if the arrays of longitudinal strain rate values obtained from the cross correlation techniques used in the preparation of L Type ST maps are integrated with respect to time, they can be used to derive displacement maps that track the lengthwise position of fixed points on the gut segment. While such maps are of little physiological interest *per se*, they can be used to systematically correct a D map so that a column of the D map corresponds to a fixed point on the segment surface rather than a certain distance along the segment i.e., to defeat shunting in D maps.

Whilst both D and L maps give an incomplete picture of the mechanical state of the muscle, recent work has identified local changes in the mechanical states of the intestinal wall during intestinal contraction on a basis of the relationship between local changes in length distinguished on D maps and concurrent local pressure within the lumen determined by fiberoptic catheters (Costa et al., 2013b). These workers were able to distinguish sites of isovolumetric and isobaric contraction and relaxation, passive shortening and elongation and auxotonic contraction and relaxation (where the muscle changes force and length simultaneously). Areas of passive dilatation and shortening contributed to <4% of the map and two dimensional plots of active muscle contraction vs. active relaxation were of broadly the same configuration as the corresponding D maps (Costa et al., 2013b). Given that the interpretation of the mechanical states of areas that abut regions of active contractile shortening was likely to be confounded to some extent by shunting, and that fine pressure resolution would be to some extent compromised by the low density of pressure sensors (one per cm), these results indicate that, despite of their shortcomings, ST maps can provide a useful overview of contractile patterns.

A number of parameters can be mathematically derived from D or L maps by application of a suitable algorithm e.g., to derive maps of frequency and velocity (Hennig et al., 2010a). A number of conventional image processing operations can be applied to the ST maps including high, low and band pass filters, mathematical operations with scalars or other images, autocorrelation, and 2D Fourier transforms (Jähne, 2004; Russ, 2006).

The difference between two maps may be mapped by simple subtraction of arrays of values on one map from the corresponding values of the other. This technique has been used to highlight the limited coordination of the ripple contractions that occur in adjacent intertaenial domains in the haustrated proximal rabbit colon (Lentle et al., 2008).

**Figure 7:**
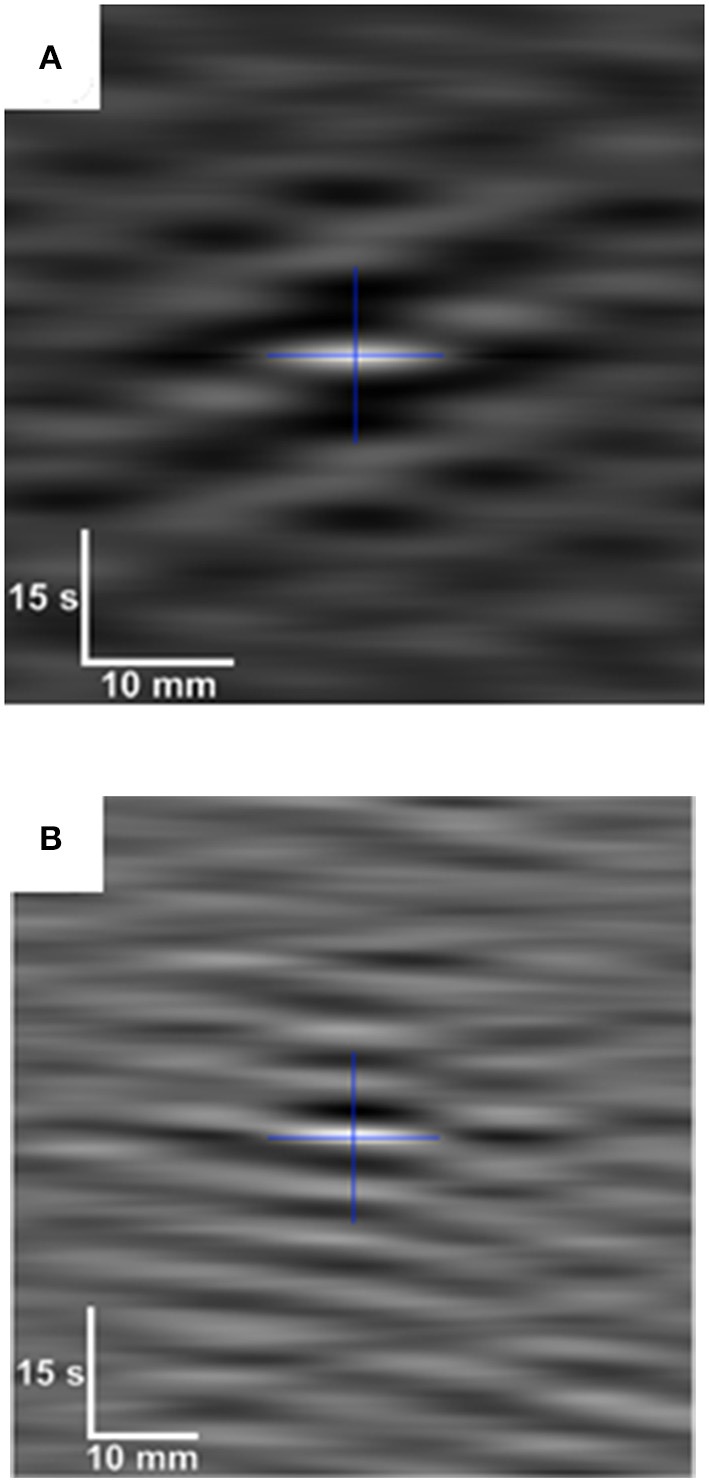
Auto-correlogram **(A)** and cross-correlogram **(B)** of data from D-type and L-type ST maps during pendular/segmentative activity in the terminal ileum of an anesthetized pig. White indicates high levels of correlation and darker shades lower correlation. The cross indicates the geometric center of each correlogram. The auto-correlogram **(A)**, which is from a subsection of an L map of ileal motility, shows a pattern in which a longitudinal contraction at a particular point along the gut occurs with every second or third wave of the base frequency (center). Hence, the variability in the spacing between lighter (contraction) bands at all points around the single central band of the base frequency, indicates local variability in contractile responses to slow waves. The cross correlogram **(B)** (bottom) is derived from synchronous subsections f of D-type and L-type ST maps of ileal motility. It shows an underlying pattern of regular spacing between contractile events at all points around the central band of the base frequency indicating that the two events likely result from a common initiating signal. Figure adapted from Janssen et al. (2014).

Again the correlation of a particular D-type or L-type ST image, with itself when it is displaced over an array of known distances in an array of known directions (i.e., autocorrelation), or with the synchronous ST image of contraction in another dimension (cross correlation), can be useful in identifying and quantifying repeating features within the map (Russ, 2006; Figure 7). The operation has the effect of averaging the individual repeating features and removing noise. The central event in the autocorrelation image represents the distance that the second image can be displaced before the features no longer lie on top of each other, and thus provides the best measure of the size of the repeating elements in the two images. Other less bright repeating events at particular angles and distances from the center of the autocorrelation image indicate correlation between the points in the original image with those that are displaced by a particular distance and direction i.e., rhythmicity. The form of the repeating elements on the autocorrelograms of ST maps can also be used to better distinguish hydraulic distension induced by regular contractions at other sites, from the direct effects of propagating contractions. Hence the repeating points generated by (instantaneous) hydraulic distension appear as parallel horizontal lines whilst those formed by (more slowly) propagating phasic contractions appear as parallel angled lines, the speed of their propagation being inversely proportional to their slope.

### Spatiotemporal maps of luminous intensity (I maps)

A further technique that is useful for tracking movements that are not necessarily related to contraction or strain is based on the detection of movement of light, reflected from or transmitted through, the tissue under study. The rows of the I-type ST map therefore correspond to the light intensities along a user-specified LOI.

The mapping of changes in patterns of light intensity is useful for the detection of the incidental movement of passive structures such as the displacement of mucosal folds as a result of changes in the internal dimensions of the lumen (Lentle et al., 2013). I mapping can also be used to track light that is generated within the preparation. Hence, the intensity map methodology can be used to track the movement of local florescence associated with contractile activity (Park et al., 2006) and the migration of electrophysiological phenomena (Lammers et al., 2001; Seerden et al., 2005) notably activation of myogenic cells such as ICCs (Spencer et al., 2007; Bayguinov et al., 2010a,b).

Where practicable concurrent L type ST mapping can be used to determine whether the movement detected by I maps is spatiotemporally coincident with the region of contraction (Lentle et al., 2016).

**Figure 8:**
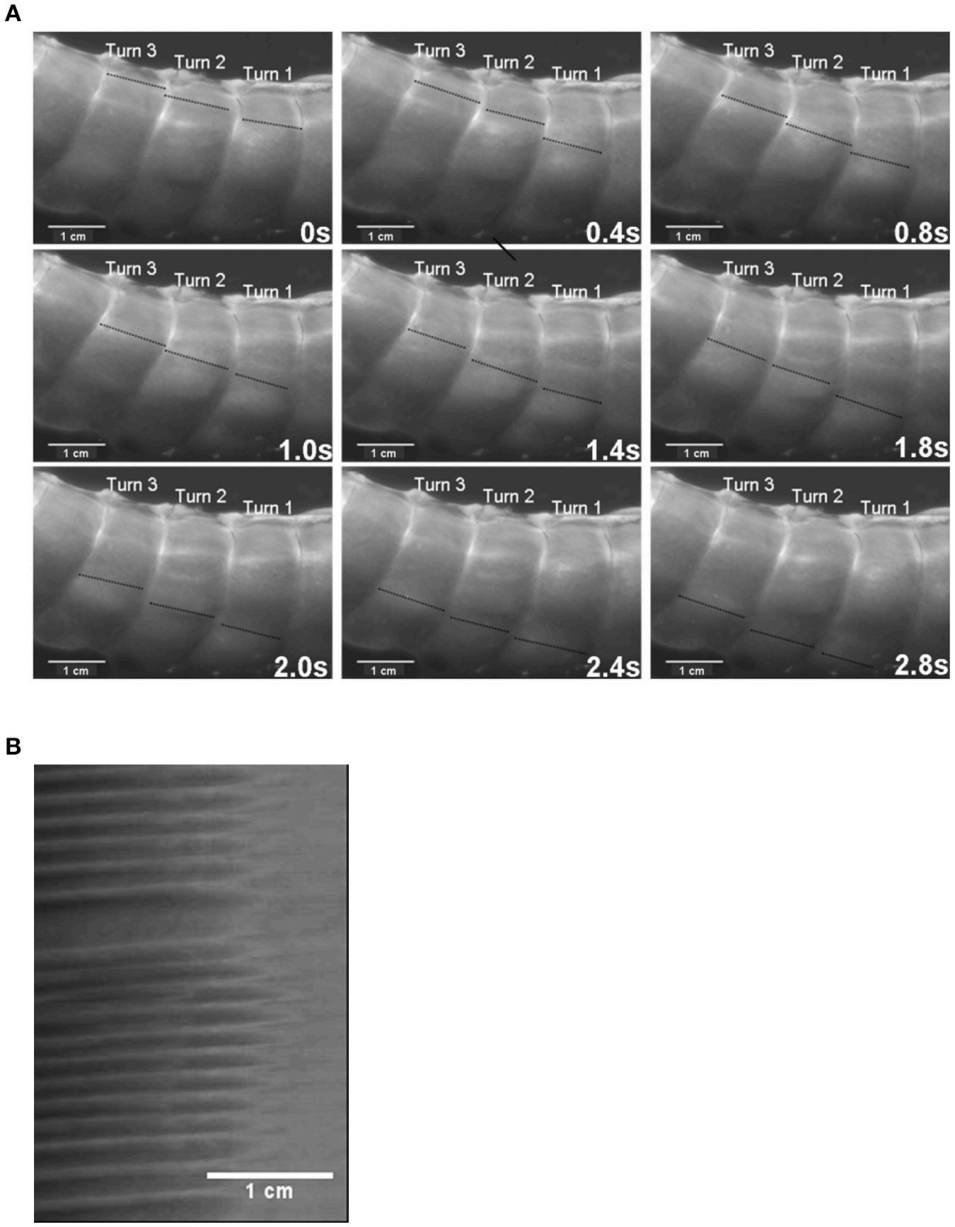
The use of I maps in determining the frequency of ladder contractions in the rabbit caecum. Photo sequence **(A)** shows a sequence of video frames of lengthwise ladder contractions (dark lines) in three successive spiral turns of the mid caecum at 0.4 s intervals. The I type ST map **(B)** is taken from of a single spiral turn during the transit of ladder contractions. The X axis represents the width of the turn (number 2 on the photo sequence) with the proximal end of the preparation to the right. Note that the arrays of contractions do not occupy the proximal third of the turn and that the slope of the largely regular sequence of contractions indicates that they are propagating from the proximal to the distal end of the turn. Figure adapted from Hulls et al. (2012).

Whilst the I mapping technique has been used to estimate the frequency and propagation velocity of contractions (Figure 8; Hulls et al., 2012) their quantification is not as reliable as that from D and strain rate maps. Where a preparation is illuminated by light from a fixed direction, displacement of the gut wall may cause the direction in which light is reflected from its surface into the camera to change. Hence light may initially be reflected from the apex of a propagating distension and subsequently from its side so that the rate of progression of the brightest region in the intensity map will not provide a true estimate of the rate at which the distended area is propagating. Again, local variation in the thickness of the gut wall through which light must propagate may influence its intensity so that it is not consistently proportional to the amplitude of the source that generates it. Hence it is always advisable to check the values obtained from intensity maps against those obtained by other means.

## Two dimensional (area) video spatiotemporal mapping

### Method and use

**Figure 9:**
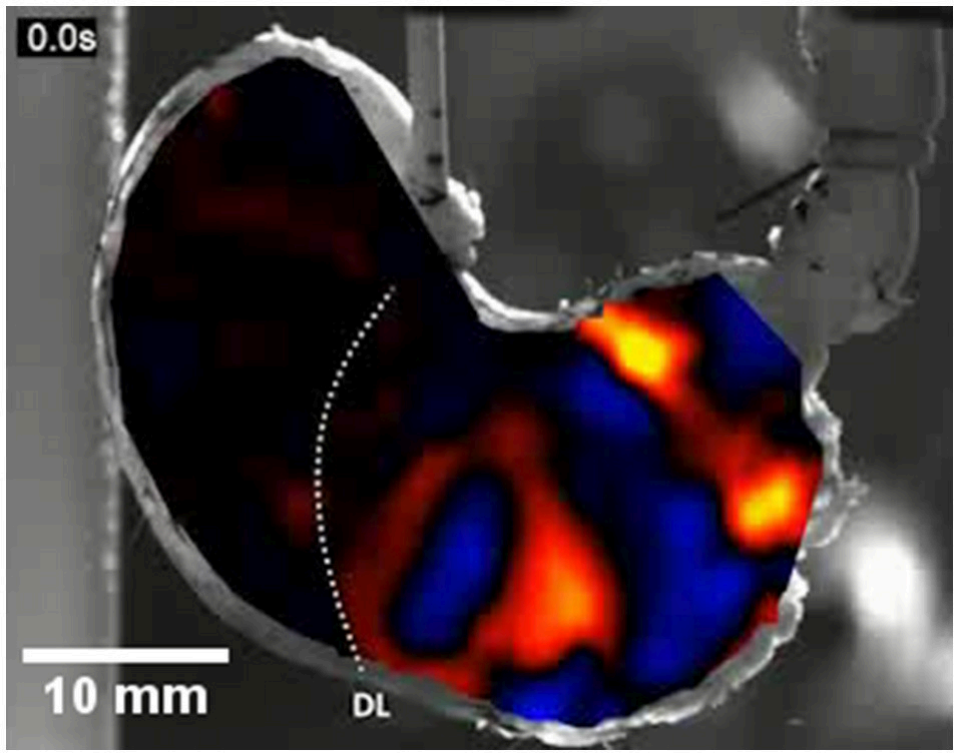
An A-type ST map of area strain rate from antral peristalsis in the stomach of the rat. The pylorus lies on the right and the (cannulated) esophagus is situated at the top slightly to right of the midpoint. The area strain rate map is overlaid onto, and synchronous with, the image from which it is derived. Note the randomly dispersed carbon particles on the areas to the side of the A-map which are also in the area under the map and were used as reference points. The succession of areas of high negative strain rate (yellow and red) are sites undergoing antral peristalses. The areas of positive strain rate (blue) correspond to areas of relaxation. The dotted line marked DL indicates the position of the line of demarcation between the fundus and body, which is anatomically evident in rats. Note the increase in the magnitude of area strain as the contractions near the pylorus and the failure of antral peristalses to extend to the lesser curve in the proximal corpus and antrum. Modified figure from Lentle et al. (2016).

The strain rate mapping technique may be extended from mapping changes in a linear dimension, i.e., along a single LOI, to map changes in areas between groups of markers that are orientated at right angles to each other, i.e., between local longitudinally and radially orientated LOIs, on successive frames (Figures 1H–J). Such a “rate of change in area” or A-type ST mapping technique is broadly similar to the “rate of change in volume” technique used in three dimensional mapping of contraction in the wall of the heart (Tops et al., 2005) save that it operates on the surface of the organ rather than the volume of the tissue. The derived maps of changes in area strain rate (A-type maps), can subsequently be overlaid onto the corresponding video image so as to provide real time record of changes in the sites of contraction (negative area strain rate) and expansion (positive area strain rate) (Figure 9).

Direct synchronous overlays of real time A-type maps also enable the shapes and extents of areas of contraction contraction (or relaxation) on the surface of an organ or compartment e.g., the body of the stomach, Lentle et al. (2010) to be determined. Again, comparison of overlays on successive frames, allows the principal direction of propagation of patches of contraction to be directly visualized and an appropriately orientated linear or curved LOI to be identified for subsequent unidimensional (L type) strain rate mapping. This can also allow circular (re-entrant) paths of contraction that may follow from disturbances in slow wave propagation associated with conditions such as gastroparesis (O'Grady et al., 2012) to be identified. The dynamics of the development and involution of contractions may also be quantified by appropriate use of algorithms that compute mean number of contractile sites, patch area and CV, perimeter to area ratio, shape index and perimeter area fractal dimension (McGarigal, 2014) on successive frames. Again the use of algorithms that sum the total areas of contraction within successive frames (core area %) can be used to assess cyclic contractile behavior and its co-variation with lumen pressure on an organ scale (Lentle et al., 2015).

**Figure 10:**
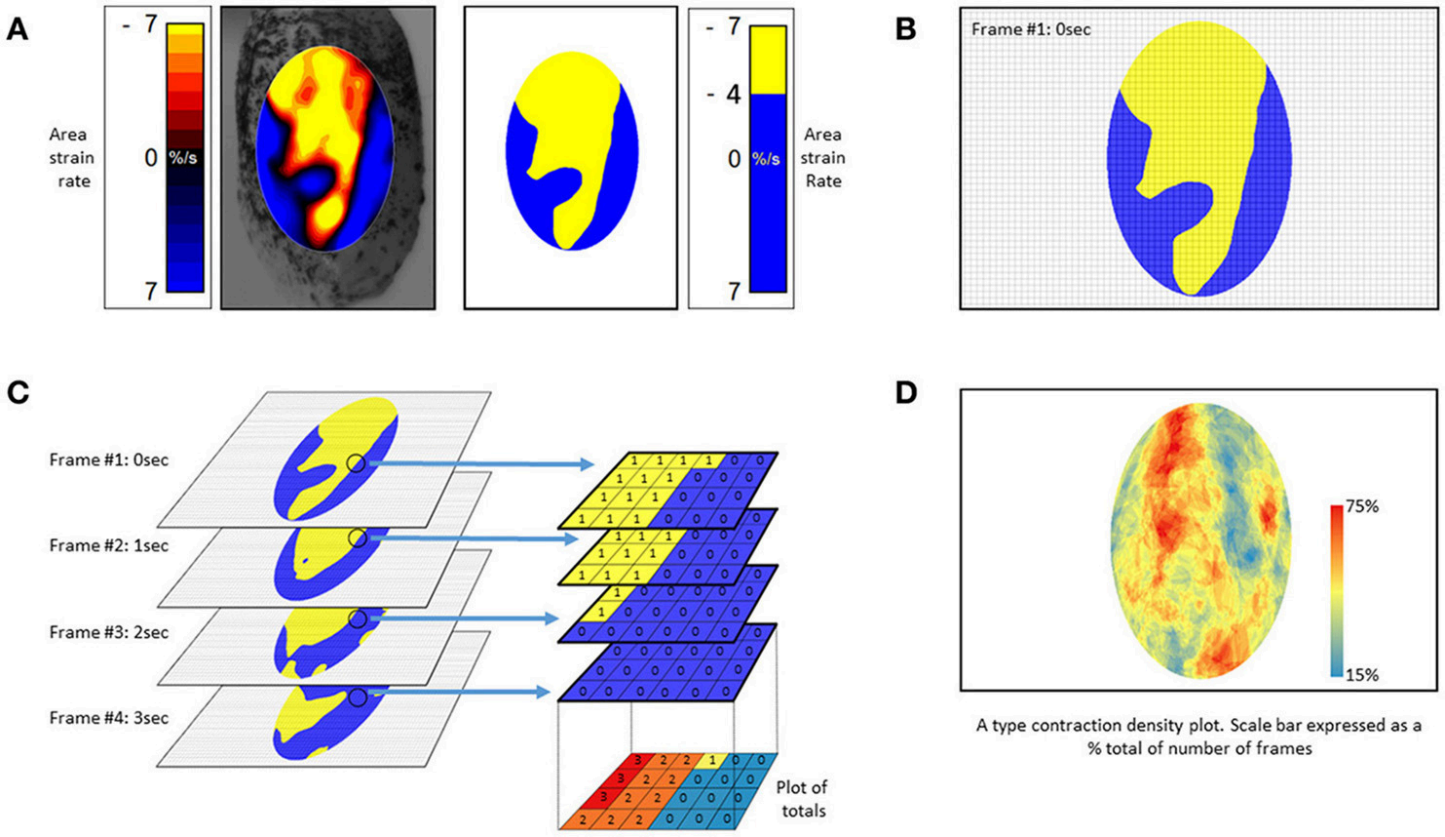
Method of construction of **(A)** type contraction density plots. **(A)** Successive Area Spatiotemporal maps are thresholded to include all sites at which strain rate exceeds a chosen value (in this case <-4% strain s^−1^). **(B)** Frames are suitably subsampled at chosen time intervals; **(C)** The subsampled frames, taken over a chosen time span, are stacked and the number of frames within each pixel of the stack with strain rates in excess of the chosen threshold counted. The results are then plotted as a color coded percentage total to produce a stacked A-type contraction density plot. **(D)** Typical stacked **(A)** type contraction density plot covering over 45% of the anterior surface of the bladder. **(B)** Adapted from Hulls et al. (2017).

The use of an algorithm that stacks successive A-type ST maps over a given period of time and sums the number of occasions that contractions, i.e., periods of negative area strain rate, occur at each pixel location allowing the effect of time to be directly incorporated into a single map (Figure 10). The resulting stacked A-type contraction density plots with color coded rates of occurrence of contractions at each pixel (Figure 10D) may be viewed as similar to the stacked sequence of the lengthwise location of contractions over time seen on D-type ST maps (Figure 2) save that the direction of propagation is visualized over 360°. Hence the highest number of contractile pixels will occur at sites where contractions repeatedly propagate or originate, the number decreasing in the surrounding zones according to the predominant direction of propagation. Such maps can also be useful in factoring out lateral expansion and determining the mean direction of propagation of a patch of contraction across a surface and hence in identifying an appropriate LOI (Lentle et al., 2015, 2016).

### Disadvantages

A type strain rate area maps and stacked A type ST contraction density plots suffer from similar disadvantages to those of linear strain rate maps i.e., L-type ST maps, in that they record only the strain rate and cannot distinguish the negative strain rate of contraction from that of elastic recovery.

## Other technical matters

The principal arbiter of high quality video spatiotemporal mapping is definition. Hence wherever possible the use of color video cameras should be avoided as the local grouping of sensor cells in arrays of three different color sensitivities decreases the overall level of resolution.

The contraction rates of gastrointestinal, arterial and urinary smooth muscle are relatively slow. Thus sampling rates of 15 frames per second, are adequate for the mapping of most phasic contractions in the GI tract. However, studies of certain neurogenic contractions such as the fast phasic contractions found in the colon (Lentle et al., 2008) may require faster rates. Again mapping algorithms may fail to detect changes in distances between surface markers in successive frames when the rate of change is low. Hence, the mapping of tonal contractions may require appropriate down sampling of the stream of video frames.

## Conclusion

The judicious use of video spatiotemporal mapping techniques has and will enable significant advances in our understanding of the physiological basis of contractile activity in the various compartments of the gut and the effects of these on the mixing and propulsion of their contents. There are nevertheless a number of limitations with which the operator should be familiar so as to avoid incorrect interpretation. The technique has great potential for the diagnostic assessment of motility during laparoscopic examination when suitable small, high definition video cameras become available.

## Author contributions

RL: conceived and was the main author of the paper, developed a number of methods described in the paper and published research based on them, edited the manuscript; CH: assisted in writing the paper and editing, and prepared the diagrams.

### Conflict of interest statement

The authors declare that the research was conducted in the absence of any commercial or financial relationships that could be construed as a potential conflict of interest.
